# Natural Concurrent Infections with Black Spot Disease and Multiple Bacteriosis in Farmed Nile Tilapia in Central Kenya

**DOI:** 10.1155/2020/8821324

**Published:** 2020-07-30

**Authors:** Daniel W. Wanja, Paul G. Mbuthia, Robert M. Waruiru, Lilly C. Bebora, Helena A. Ngowi

**Affiliations:** ^1^University of Nairobi, College of Agriculture and Veterinary Sciences, Department of Veterinary Pathology, Microbiology and Parasitology, P.O. Box 29053-00625, Kangemi, Nairobi, Kenya; ^2^Animal Health and Industrial Training Institute (AHITI) Kabete, P.O. Box 29040-00625, Kangemi, Nairobi, Kenya; ^3^Sokoine University of Agriculture, College of Veterinary and Medical Sciences, P.O. Box 3000, Chuo Kikuu, Morogoro, Tanzania

## Abstract

Nile tilapia (*Oreochromis niloticus*) is the most cultured and available fish for Kenyan consumers, and therefore, any tilapine disease deprives them the valuable source of protein. Nile tilapia farm was diagnosed with severe concurrent black spot disease and multiple bacteriosis using gross lesions and parasitological, histopathology, and standard bacteriological procedures. A total of 25 fish were sampled and inspected, and all of them had raised, macroscopic 1 mm-sized black spot lesions. The mean intensity of black spots per fish was 728 with an abundance of 2–1740 metacercariae cysts per fish. A high intensity of black spot infestation was observed in the fins (43.9%), skin and underlying muscles (18.3%), and gills (18%). In addition, histopathological data confirmed presence of a metacercaria of *Neascus* spp. as the aetiological agent of black spot disease. Furthermore, a thick fibrous capsule around the metacercaria, black pigment melanomacrophages, and moderate muscle atrophy were observed. The most prevalent bacteria isolated were *Aeromonas*, *Enterobacter cloacae*, *Klebsiella pneumoniae*, and *Micrococcus luteus*. Physicochemical parameters of pond water were temperature (28.2°C), dissolved oxygen (4.2 mgl^−1^), pH (8.5), ammonia free nitrogen (15.8 mgl^−1^), alkalinity (112 mgl^−1^), hardness (68 mgl^−1^), nitrites (0.058 mgl^−1^), nitrates (58 mgl^−1^), and phosphates (0.046 mgl^−1^). However, the levels of nitrates, nitrites, alkalinity, and ammonia free nitrogen exceeded the recommended limits. In conclusion, these findings suggest that coinfections by these organisms coupled by water quality-related stress can be associated with low-grade mortality observed in postfingerling tilapia as well as reduced growth. The authors recommended immediate destocking, thorough disinfection, and control of piscivorous birds. Moreover, attention ought to be geared towards prevention of parasitic infestations in fish so as to minimize fish deaths related to secondary bacteriosis. Further experimental studies should be carried out to elucidate the relationship of these pathogens.

## 1. Introduction

Nile tilapia (*Oreochromis niloticus*) is a resilient species, which grows in a diverse range of aquatic environment, enduring extreme limits of dissolved oxygen, temperature, and contaminants [1]. Nile tilapia possesses advantageous characteristics for pisciculture, including ability to grow rapidly [2], excellent feed conversion efficiency [3], high resistance to disease [4], and ability to reproduce in captivity [5]. This justifies the dominance of Nile tilapia in global-scale production. The roughness and/or resilient nature and disease resistance make it an ideal aquatic “zebu”. However, recent epidemics such as tilapia lake virus have threatened the species and consequently have outlined the importance of protecting the species through increased disease surveillance [6].

Farmed tilapine fish are exposed to single or multiple pathogens such as parasitic, bacterial, or mixed infections leading to diseases and mortalities [7]. Even though multiple or concurrent infections are common naturally, most studies on tilapia diseases focus on isolation of single pathogen [8]. Poor understanding of coinfections may explain this observation [9].

Bacterial diseases constitute the most important infections threatening sustainability of intensively farmed tilapia [10, 11]. Common bacterial diseases with an enormous economic effect on farmed Nile tilapia in Kenya include motile aeromonads septicaemia, streptococcosis, and pseudomonads septicaemia [12–14]. On the other hand, parasites are the basis of all fish epidemics that are associated with secondary invasion with bacteria [15, 16] and can act as a vector to transmit bacterial pathogens [15, 17]. Parasites affecting Nile tilapia under Kenyan aquaculture include myxozoans, monogeneans, nematodes, digenean trematodes, acanthocephalans, and cestodes [18, 19].

Black spot disease or black grub disease is caused by the metacercarial stage of several genera of digenean flukes among the families *Heterophyidae* and *Diplostomatidae* [20]. Species of the genera *Apophallus*, *Crassiphiala*, *Uvulifer* [21], *Bolbophorus* [22], and more generally as *Neascus*-type trematodes [23, 24] have been documented in many freshwater and saltwater fish. The pathogenesis of black spot disease follows penetration of larvae forms (cercariae) of the parasites into the skin of a fish, where they encyst and develop into metacercariae [25]. The metacercariae provokes the host to form a fibrous capsule around the parasite. The host immune and/or inflammatory response results into infiltration of melanomacrophages through the fibrous wall of the cysts, hence causing the typical appearance of black spots [26]. The black spot lesions are usually visible grossly.

The objective of this study was to describe the occurrence of natural coinfection of black spot disease with multiple bacteriosis in Nile tilapia (*Oreochromis niloticus*) in a small-scale farm in Central Kenya that was experiencing mortality and aesthetic rejection of fish.

## 2. Materials and Methods

### 2.1. Study Area and Case History

The study was conducted between the months of February and November 2018, in a farm located in Kibingo, Kirinyaga Central subcounty, Central Kenya, on longitude 37°15.256E and latitude 00°29.085S and at an altitude of 1652 m above sea level. The farm owns a single 336 m^2^ sized ultraviolet-treated plastic-lined pond, stocked with mixed sex Nile tilapia. The disease history was obtained from the owner. The initial fish stocking density was 1000 fingerlings; however, current stocking density was unknown due to inbreeding, partial harvests, and mortalities. The disease was surmised when the owner noted raised black skin lesions on fish with low mortality (especially the postfingerlings) and high morbidity and harvested fish rejected by consumers.

### 2.2. Water Quality Assessment

Water parameters such as temperature, dissolved oxygen, and pH were measured *in situ* using waterproof handheld HANNA Multiprobe meters 9142 and 98127, respectively (Hanna Instruments Inc., USA), at three different sites on the pond. Water samples were collected and analysed for ammonia free nitrogen, alkalinity, hardness, nitrites, nitrates, and phosphates following standard methods for examination of water and wastewater developed by Boyd and Tucker [27].

### 2.3. Fish Sampling, Necropsy, and Parasitological Examination

Following owner's consent, twenty-five fish were harvested using a seine net. These were then transported alive in buckets with source water to County Veterinary Laboratory, Kerugoya, for necropsy and analysis.

Postmortem examination was performed using standard procedures as described by Noga [28] and Roberts [29]. The fish were stunned with a single blow to the back of the head and pithed to separate the central nervous system from the spinal cord. An external examination of individual fish was performed to check for occurrence of ectoparasites and gross lesions. Prior to dissection, individual fish weight (g) and total length were recorded for calculation of the condition factor. The condition factor was calculated by a formula given by Froese [30]:  *K*=(*Wx*100/*L*^3^), where “*K*” is Fulton's condition factor, “*W*” is the wet weight in grams, and “*L*” is the total body length in centimetres. The sex of the fish was also recorded.

### 2.4. Histopathological Examination

Tissue specimens were taken from the skin and underneath muscle from the flanks of few *O*. *niloticus* that were heavily infested with black spot lesions. The samples were fixed in 10% buffered formalin overnight. The tissue sections were then gradually dehydrated in 70–100% ethanol, cleared in xylene, and finally embedded in paraffin wax through standard procedures [31, 32]. Tissue specimens were sectioned at 3–5 *µ*m and stained with haematoxylin and eosin (H&E). Slides were then observed under a light microscope.

### 2.5. Bacteriological Examination

Out of the 25 fish collected, 10 fish were randomly selected for bacteriological isolation, which was done on aseptically collected kidney swab from individual fish. These swabs were streaked separately and aseptically onto plates containing nutrient agar, 10% sheep blood agar, and MacConkey agar. The inoculated plates were incubated aerobically at room temperature (24°C–26°C), in an inverted position. After 24–48 hours of incubation, the plates were examined, and colony morphology on the plates was recorded. The isolates were identified using colony morphology, Gram staining characteristics, conventional biochemical tests (catalase reaction, cytochrome oxidase, methyl red, citrate utilization, urea degradation, sulphur-indole-motility, and sugars fermentation) following Austin and Austin [11], Bergey's Manual of determinative bacteriology [33], and Markey et al. [34]. Further characterization of Gram-negative lactose fermenters was conducted using the Analytic Profile Index 20E (API 20E) microbial identification strips according to manufacturer's protocol (BioMérieux Marcy-l'Étoile, France).

### 2.6. Data Analysis

The disease prevalence, mean abundance (average number of black spot lesions seen in all the fish), and mean intensity (mean number of black spot lesions per fish infested) were calculated as described by Bush et al. [35]. The collected data were validated, entered, and stored in Microsoft Excel® spreadsheet, which was also used to calculate means and proportions. Inferential statistics were performed using Statistical Package for the Social Sciences (SPSS®), version 22.0. The chi square test was used to compare proportions. Pearson correlation matrix was used to check for association. All tests were tested at a level of 0.05 for significance.

## 3. Results

### 3.1. Physicochemical Parameters of Pond Water and on Farm Observations

The pond water colour was dark green, an indication of overfertilization. The average values of other parameters measured are summarized in Table 1. From the table, the pond water temperature, pH, dissolved oxygen, water hardness, and phosphates levels measured were within the recommended/desired range for rearing tilapia. However, levels of nitrates, nitrites, alkalinity, and ammonia free nitrogen were above the recommended limits.

### 3.2. Fish Samples and Their Biodata

Of the 25 fish sampled, 16 were females and the rest were males. The mean weight, standard, and total lengths of the fish samples were 119 ± 4.7 g, 15.2 ± 0.2 mm, and 19 ± 0.3 mm, respectively. The mean condition factor (*K*) of fish was 1.73 ± 0.03, with no significant difference between males and females.

### 3.3. Macroscopic and Necropsy Findings

**Figure 1:**
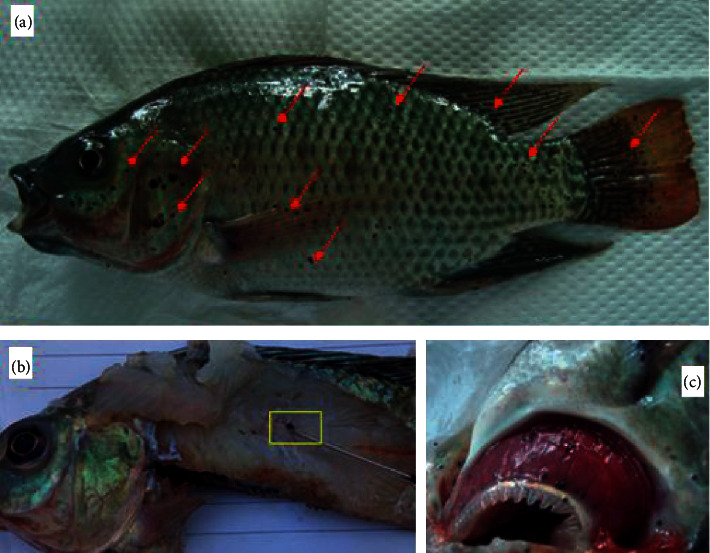
A Nile tilapia sampled from the farm showing multiple black spot lesions on the body (a). Black spot lesion embedded in the muscle (b) and on the gills rakers and filaments (c) of *Oreochromis niloticus*.

On macroscopic examination, all the 25 fish examined were infested with macroscopic 1 mm raised black spot lesions, with no other apparent clinical signs (behavioural) of parasitism or bacteriosis. The lesions were extensively distributed throughout the body surface including the fins, operculum, mouth, skin, gills, and eyes (Figure 1(a)). On dissecting the fish, more conspicuous black spots were also observed on the epidermis, dermis, and musculature and on the gill rakers and filaments of fish (Figures 1(b) and 1(c)). There were lesions in the vertebral bone; however, no skeletal deformity was observed. There were no significant findings seen in other visceral organs.

Table 2 shows distribution of lesions in various organs and their corresponding percentage. The fins were the most infested. The mean intensity of black spots per fish was 728.48 3 with an abundance range of 2–1740 metacercariae cysts per fish. There was a significant negative correlation between intensity and body condition (*R*^*2*^ = −0.49).

### 3.4. Histopathological Findings

**Figure 2:**
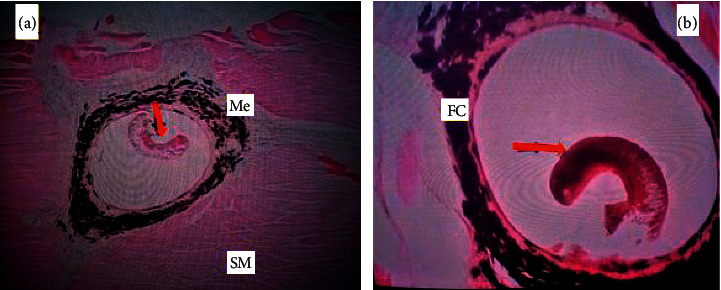
Photomicrographs of encysted metacercaria of *Neascus* spp. (red arrow). The fish surrounds the cyst with black pigmented melanin produced by melanomacrophages in response to the parasite. FC, fibrous capsule; Me, melanomacrophages and pigment; SM, muscle tissue.

Histopathological findings revealed that the aetiological agent causing this disease was a metacercaria of *Neascus* spp., a digenean trematode. The parasite localized in the stratified epithelial tissue and musculature (Figure 2(a)). Histopathological analysis further revealed a thick fibrous capsule around the encysted metacercariae with the periphery of the cyst containing black pigment due to melanomacrophages responsible for the formation of dark spots (Figure 2(b)). Moderate muscle atrophy was also observed.

### 3.5. Bacteriological Findings

**Figure 3:**
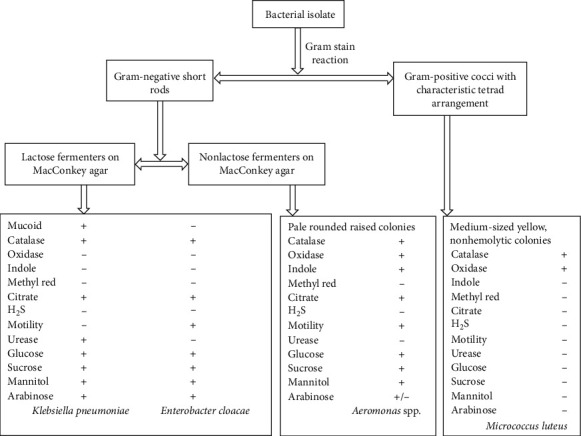
Schematic diagram showing Gram stain reaction, colony morphology, and some biochemical tests used to identify various bacterial isolates.

The results of bacteriological examination revealed 4 bacteria taxa, which were identified by colonial morphology, Gram stain reaction, and by use of conventional biochemical tests (Figure 3). The predominant bacteria were *Aeromonas* spp. (30%, *n* = 3), *Enterobacter cloacae* (20%, *n* = 2), *Klebsiella pneumoniae* (10%, *n* = 1), and *Micrococcus luteus* (10%, *n* = 1), while 30% (*n* = 3) of the kidney swabs yielded no bacterial growth with respect to isolation procedures used.

## 4. Discussion

Although black spot disease (BSD) does not cause much pathological effect, it affects the aesthetic appeal of fish, therefore causing rejection at market level [25]. However, the black spots can be quite pathogenic if they are located in sensitive areas such as gills. Gill infestation leads to respiratory distress [39], while fish with eye infestation may be blind.

Coinfections occur when hosts are infected by two or more different pathogens either concurrently or as secondary invaders, so that two or more pathogenic agents are active together in the same host [40]. This study confirmed such coinfections of metacercarial stages of *Neascus* spp. causing “black spot” disease with several bacteria genera including *Aeromonas*, *Enterobacter*, *Klebsiella*, and *Micrococcus* in farmed Nile tilapia collected from Kirinyaga County. The results are contrary to a previous study in Winam Gulf of Lake Victoria, Kenya, by Thon et al. [41] who reported a prevalence of 0.7% in wild Nile tilapia. The differences in prevalence rate may be due to the fact that parasitic infestations might be annihilating in farmed fish compared to wild fish [42]. This is so because farmed fish is associated with stressful culture conditions including overcrowding and poor water quality, thus making them more susceptible to diseases [42, 43]. Moreover, Thon et al. [41] focused on parasitological isolation of endohelminths of wild Nile tilapia, and therefore, the bacteriological status of the sampled fish was not investigated. The findings are, however, partially in line with a previous study in Pacific Northwest by Arkoosh et al. [23] who reported coinfection of black spot disease and *Renibacterium salmoninarum* in juvenile salmon (*Oncorhynchus* species). In this study, several bacteria taxa including *Aeromonas* spp., *Klebsiella pneumoniae*, *Enterobacter cloacae*, and *Micrococcus luteus* were isolated from the kidney of examined fish. The discrepancy in bacteria taxa in the current study and that of Arkoosh et al. [23] could be due to culture environment of the fish species; tilapia is a warm water species, while salmon is a cold water fish. The bacteria isolated in this study are ubiquitous in aquatic environments and have been reported previously in Kenya [13, 14]. Some of these including *Aeromonas* spp. and *Klebsiella pneumoniae* are pathogenic to fish [11], while *Micrococcus luteus* have been developed as fish probiotics [44]. Recovery of these bacteria from the kidney is an indication of infection after overcoming the fish defence mechanisms.

In this study, all the sampled fish had a condition factor of more than one. A condition factor value of more than one implies a good fish health condition and proportional growth, which is recommendable in a fish farm [45]. However, histopathological sections revealed moderate muscle atrophy; which explains the negative correlation between parasite intensity and condition factor. This perhaps suggests that this was a case of recent infection, considering that none of the fish had skeletal abnormality.

Physicochemical parameters of the water and nutrients and presence of pollutants have a positive influence on the occurrence of parasitic and bacterial populations and communities in fish cultured environments [46–49]. The fish pond recorded slightly high levels of calcium carbonates (alkalinity) and high levels of nitrites, nitrates, and ammonia free nitrogen than the recommended range for rearing tilapia. A study by Ismail et al. [50] demonstrated that presence of *Aeromonas hydrophila*, *Enterobacter cloacae*, and *Micrococcus* spp., among other bacteria was greatly influenced by water temperature, levels of nitrites, phosphates, sulphide, and ammonia. On the other hand, Isyagi et al. [36] reported that consistently high levels of ammonia nitrogen above the recommended limit are associated with trematode infestations such as *Neascus* spp. As such, poor water quality may reduce fish immunological ability and thus become more susceptible to disease-causing pathogens [48], including bacteria and parasites.

This could be the first report on natural concurrent infection of *Neascus* spp. and multiple bacteriosis. However, the relationship among these pathogens is not fully understood. In a probably similar pathogenesis cascade as the current study, Sandell et al. [51] reported stunted growth and low survival chances among juvenile salmons due to concurrent infections of *Neascus* spp. and *Renibacterium salmoninarum*. Some researchers have postulated that coinfections of some ectoparasites simultaneously with ubiquitous bacteria have a mutual benefit relationship [52, 53], unlike in this case where there were mortalities. A number of studies have shown synergetic interaction of parasitic and bacterial coinfections [54, 55]. These studies have shown higher mortality rates in concurrent bacterial-parasitic infections. This synergistic effect has been explained as a result of stress caused by parasites that lowers fish immunity to other infections including secondary bacterial infections [56], as well as the damaging effects caused by the parasite that provide portal of entry for the secondary invaders including bacteria.

Due to lack of prior studies on coinfections in fish, particularly in farmed Nile tilapia, we can compare with findings from other systems on parasite species from the same fish in Kenya and beyond. For instance, Florio et al. [57] reported a slightly higher parasitic infestation of 86.5% in pond-cultured tilapia compared to wild or caged fish in Kenya. Florio et al. [57] reported 23 different parasite genera in Kenya, 15 in Uganda, and 13 in Ethiopia under different aquatic systems. *Trichodina* spp. *Trichodinella* spp., *Myxobolus* spp., *Dactylogyrus* spp., *Gyrodactylus* spp., diplostomatid and clinostomid metacercariae, *Neascus* spp., cestodes, and larval nematodes were reported in all the three countries. Florio et al.' [57] work shows that parasitic infestation in fish vary greatly from one system to another, and this may be influenced by physicochemical properties of culture water and occurrence of intermediate hosts. Moreover, climatic/topographical conditions of the region, seasonality, and host parasite relationship may also play a role in the epidemiology of parasites of fish.

## 5. Conclusion

In conclusion, our findings show that synergistic coinfections by metacercariae of *Neascus* spp. and multiple bacteria taxa coupled with poor water quality were responsible for the reported mortality in postfingerling Nile tilapia. The findings of this study may be of significance to aquaculture, especially at a time of scanty information on diseases of farmed fish in developing countries, including Kenya.

## 6. Recommendations

It ought to be noted that presence of snails and piscivorous birds is one of the risk factors that propagate the life cycle of the *Neascus* spp. While there are no practical means to treat BSD, the authors recommend immediate destocking, thorough disinfection (including use of molluscicides), and controlling piscivorous birds. Moreover, attention ought to be given towards prevention of parasitic infestations in fish so as to minimize fish deaths related to secondary bacteriosis and economic loss due to aesthetic reasons. Further experimental infection challenge studies are recommended in order to evaluate the associations of these organisms so as to comprehend their significance on fish.

## Figures and Tables

**Table 1:** Mean values of physicochemical parameters of pond water.

Physicochemical parameters of water	Recorded pond value
(1) Temperature (°C)	28.2^c^
(2) Dissolved oxygen (mgl^−1^)	4.2^c^
(3) pH	8.5^c^
(4) Total ammonia free nitrogen (mgl^−1^)	15.8^a^
(5) Alkalinity (mgl^−1^)	112^a^
(6) Hardness (mgl^−1^)	68^c^
(7) Nitrites (mgl^−1^)	0.058^a^
(8) Nitrates (mgl^−1^)	58^a^
(9) Phosphates (mgl^−1^)	0.046^c^

Note. The recommended range of physicochemical parameters of water for culturing Nile tilapia in ponds are temperature = 22–32°C; dissolved oxygen = 5–7 mgl^−1^; pH = 6.5–8.5; ammonia 0.3–2 mgl^−1^; alkalinity 20–100 mgl^−1^; hardness 10–100 mgl^−1^; nitrite = <0.02 mgl^−1^; nitrates = 0.1–4.5 mgl^−1^, and phosphorous = 0.01–3 mgl^−1^ [36–38]. Key: a = pond value was above the recommended range; b = pond value was below the recommended range; and c = pond value was within the recommended range.

**Table 2:** Percentage distribution of black spots in various organs of infested fish.

Organ	Total number of black spot lesions	Distribution (%)
Fins	7994	43.9
Skin and muscles	3334	18.3
Gills	3286	18
Operculum	1594	8.8
Head	1012	5.6
Eyes	514	2.8
Buccal cavity	478	2.6

## Data Availability

Data associated with this paper are retrievable from the online repository at http://doi.org/10.17632/w2h8mhy3f5.2.
